# An effective behavior recognition method in the video session using convolutional neural network

**DOI:** 10.1371/journal.pone.0266734

**Published:** 2022-08-01

**Authors:** Yizhen Meng, Jun Zhang

**Affiliations:** Computer Science Department, Tangshan Normal University, Tangshan, China; University of Engineering & Technology, Taxila, PAKISTAN

## Abstract

In order to further improve the accuracy of the video-based behavior recognition method, an effective behavior recognition method in the video session using convolutional neural network is proposed. Specifically, by adding the target detection phase before the behavior recognition algorithm, the body region in the video can be accurately extracted to reduce the interference of redundant and unnecessary background noises, and at the same time, the inappropriate images can be replaced, which has reached the role of balance background trade-off, and finally, the neural network can learn the human behavior information with emphasis. By adding fragmentation and stochastic sampling, the long-time time-domain modeling of the whole video session can be established, so that the model can obtain video-level expression ability. Finally, the improved loss function is used for behavior recognition to solve the problem of classification difficulty and possible sample imbalance. In addition, we conducted the hyperparametric experiment, the ablation experiment and the contrast experiment on different open source and benchmark datasets. Compared with other commonly used behavior recognition algorithms, the experimental results verify the effectiveness of the proposed method. In addition, the related deep learning-based methods used in behavior recognition are reviewed at the beginning of this paper, and the challenges in behavior recognition and future research directions are prospected at the end of this paper, which will undoubtedly play a double role in the work of later researchers.

## Introduction

With the development of Internet technology and the popularity of video acquisition equipment, video has become the main carrier of information [1]. In the current era of explosive growth of video data, how to analyze and understand the content of video is becoming more and more important [2]. Automatic analysis, retrieval and recognition of human behavior in the video session is an important task in computer vision [3]. Meanwhile, it is an interdisciplinary research topic of machine vision, pattern recognition and artificial intelligence, and has been widely used in video surveillance [4], human-computer interaction [5], intelligent robot [6], virtual reality [7] and other fields. However, due to the high complexity of human behaviors and the variety of scenes, behavior recognition has become a very challenging subject [8].

**Fig 1:**
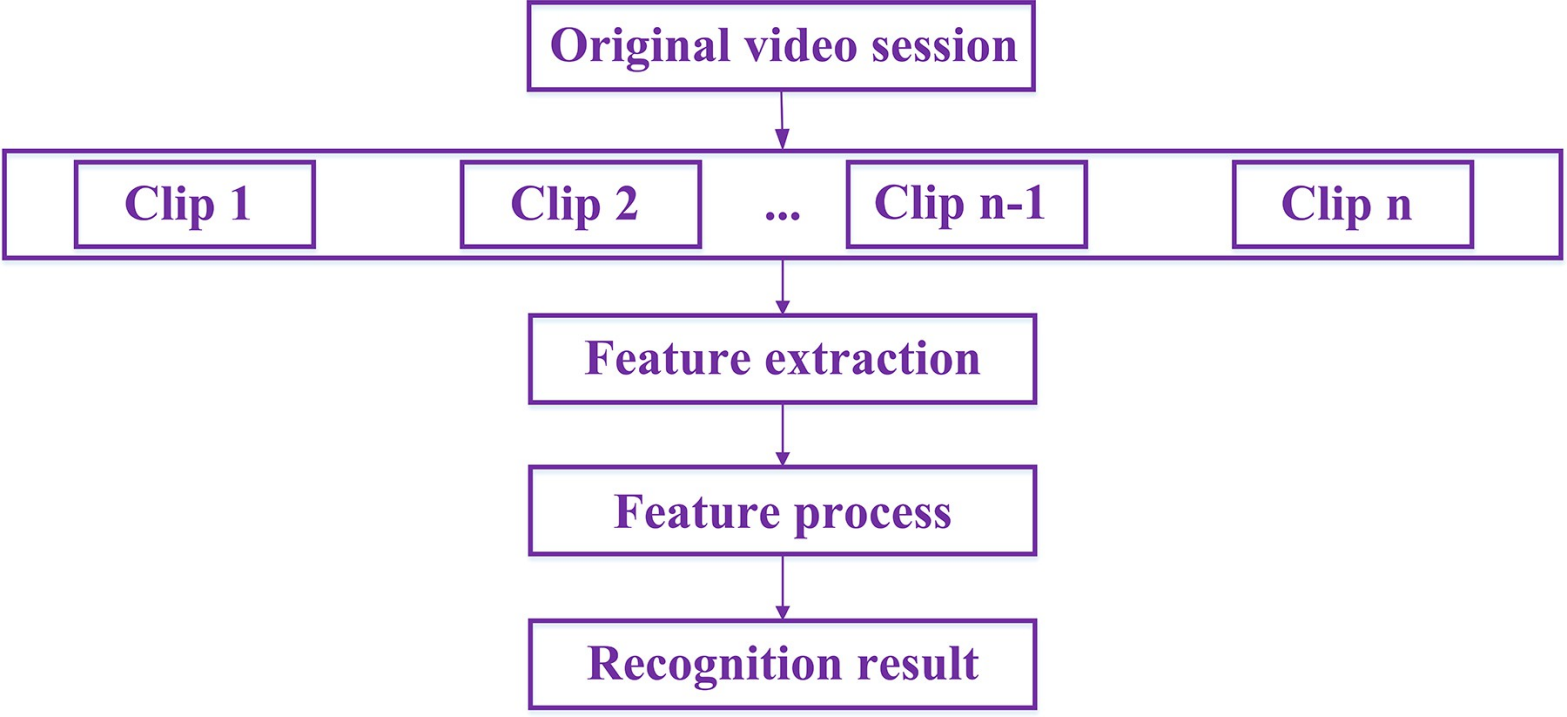
The general flowchart about behavior recognition.

In the abstract, the processing procedure of human behavior recognition in video session is mainly divided into the following four parts [9, 10]: 1) Clipping video frames from the original video; 2) Detect motion information in each video frame and extract underlying features with descriptors; 3) Conduct modeling research on behavior patterns; 4) Establish the corresponding relationship between behavior categories and low-level visual features or other high-level semantic information. The general flowchart can be shown in Fig 1. In addition, for the case that the input dimension of some learning algorithms is fixed but the feature descriptor is not fixed, it is necessary to aggregate the feature descriptors in a certain way to make the input dimension fixed.

Thanks to the development of *Big-Data* and the strong representation capability and superior performance of convolutional neural network (CNN) in the field of image processing [10], the current methods of human behavior recognition based on deep learning have outperformed the classical methods based on hand-crafted features and achieved remarkable results [11]. The deep learning-based behavior recognition method classifies the behavior in the video by learning the behavior surface autonomously through the network in an end-to-end form [12].

Because the general CNN cannot model time information enough [10], Diba et al. [13] first proposed a two-stream CNN composed of spatial stream network and temporal stream network, in which the former takes a single frame RGB image as input for modeling appearance features, and the latter uses stacked optical flow images as input to model motion features. During training, spatial stream network and temporal stream network are trained separately. During testing, softmax scores of the two streams are summarized by averaging all sampled video frames to obtain video-level prediction results. In another classic work, Karpathy et al. [14] fed low-resolution RGB frames and high-resolution center crops to two separate streams to speed up the computation. Different fusion strategies were investigated to model the temporal dynamics in videos. In addition, there are some methods to extended the two-stream CNN architecture, either by changing the fusion mode of two-stream network or changing the feature coding mode of two-stream network, that is, encoding frame-level features into video-level features[10, 15].

Later studies also showed that the fusion of RGB and optical flow methods can improve the accuracy on the test set [10]. Russakovsky et al. [16] proposed a multimodal fusion method combining RGB images, optical flow and audio information in order to improve the accuracy of the model, which is slightly more accurate but very time-consuming and takes up extra memory of the computer. Therefore, Du et al. [17] proposed a new network structure based on 3-dimensional-CNN to encode both spatial and temporal information of video in a single network, while 3-dimensional-CNN is more computationally intensive compared to 2-dimensional-CNN. However, 3-dimensional convolution will introduce a large number of parameters, which greatly increases the temporal and spatial complexity of the network, resulting in the consequences of memory consumption and sharp increase in computation [18].

In order to reduce the complexity of spatio-temporal fusion, Kumawat et al. [19] proposed a mixed 2-dimension/3-dimension convolution tube (MiCT). MiCT shares spatial information through 2-dimensional convolution and 3-dimensional convolution, and promotes it by means of 2-dimensional convolution and cross-domain residual connection learning of 3-dimensional spatio-temporal features. MiCT makes the feature mapping at each spatio-temporal level deeper before spatio-temporal fusion, so that the network can obtain better performance in fewer spatio-temporal fusion. Compared with 3-dimensional CNN stacked layer by layer, MiCT integrates 2-dimensional convolution and 3-dimensional convolution, which not only enhances feature learning, but also greatly reduces the complexity of spatio-temporal fusion. In addition, Sarabu et al. [20] proposed an Efficient Convolutional Network (ECO) architecture, which includes two parts: one is the underlying 2-dimensional CNN, which is used to model spatial features; The other part is the parallel branch of 2-dimensional CNN and 3-dimensional CNN, which is used to process the output of the underlying 2-dimensional CNN. The parallel 2-dimensional CNN branch modeling spatio-temporal features can simplify the processing and ensure that the static image features get necessary attention, while 3-dimensional CNN is mainly responsible for processing more complex actions. In the inference stage, ECO only uses a set of video frames trimmed by the center for predetection, so the memory consumption is low. However, ECO sacrifices low-level time modeling to improve efficiency, but before time fusion occurs, much useful information is lost in the feature extraction process. Subsequently, Zhang et al. [2] proposed SlowFast Networks (SFN) to solve the problem that there are differences in the evolution of temporal and spatial features of video signals. The slow path runs at low frame rate and is used to capture spatial semantic information provided by sparse video frames, and the fast path run at high frame rate to capture rapidly changing motion information at fine temporal resolution. Meanwhile, the fast path and the slow path realize information interaction through horizontal connection. Fast path can reduce the spatial resolution of input frame and remove color information, so that it pays more attention to the information of time dimension. In addition, the complexity of the model is reduced by reducing the channel capacity of the fast path.

In the traditional two-stream CNN structure, different types of information (i.e. space and time) are learned from the input video through two independent networks, and then the final result is obtained through fusion, which enables the traditional 2-dimensional-CNN to effectively process the video data and achieve the high accuracy. However, optical flow can only represent the motion information between adjacent frames, so the two-stream network has very limited access to the time context, which is not conducive to modeling some actions with a large time span, that is, there are limitations in the effective modeling of video-level time information. Fortunately, the time series modeling network, such as Recurrent neural network (RNN), Long ShortTerm Memory (LSTM) and Temporal Segment Network(TSN), can compensate for this defect.

Compared with general feedforward networks, RNN have the capacity to store information and process ordinal data. In addition, RNN have the memory of input information and it could reflect the relationship of time series data. However, when solving the long sequence problem, RNN is prone to the vanishing gradient problem. In order to improve this phenomenon, Hochreiter et al. [21] extended RNN to LSTM, replacing neurons with memory cells. In LSTM, the input gate determines the current input information to be retained, the output gate determines the information to be output to the hidden layer at the next moment, and the forget gate determines the information to be discarded at the last moment. Hence, LSTM realizes the overall update of the system state and the output of results, and it has a good effect on learning features of long sequence data [22]. Correspondingly, in order to solve the problem of two-stream network’s weak modeling ability for long-term structure in behavior recognition domain, Ng et al. [23] adopted LSTM to aggregate the CNN bottom output of video frame sequence, which can more effectively express the video frames on the time series of dependencies, so as to realize the long time series modeling. Because LRCN (Longterm Recurrent Convolutional Networks) which combined of convolution function and long time recursion could directly map the input of variable length to the output of variable length and could better obtain local temporal and spatial features, the difference between adjacent frames is taken as input in [24], and LRCN is used for encoding, thus achieving end-to-end detection of violent behavior based on video. In addition, in order to make full use of the spatial correlation of videos, Li et al. [25] proposed a movement-based attention mechanism to guide attention to relevant spatio-temporal positions.

In order to account for the long-term time-domain information in the video, there is another solution. Song et al. [26] proposed temporal segment networks (TSN). TSN introduced a sparse sampling strategy based on two-stream network. Firstly, the input video was segmented into *k* segments, and then a frame was randomly sampled from each segment, and each frame was independently extracted through CNN. The output of each segment is combined with the segment consensus function to obtain the consensus of category assumptions among segments. Finally, category scores of spatial stream and temporal stream are fused to obtain video-level prediction results. The sparse sampling strategy of TSN ensures that the input frame covers each time period in the video. This video-level supervision method enables the network to extract the global temporal and spatial features, which effectively solves the problem that the traditional dual-stream network lacks the ability of long-term structure modeling. In addition, Sun et al. [10] provided a comprehensive review about human behavior recognition methods based on various data patterns, including fusion-based frameworks and collaborative learning-based frameworks.

One of the difficulties of the existing behavior recognition is that the behavior of the target only accounts for a small part of the long video, and the moving target is interfered by a large amount of background information. Therefore, the key to behavior recognition is to extract the effective temporal and spatial information of the behavior from the long video and effectively distinguish the mixed information of the temporal and spatial background. In order to solve this problem and to make the CNN learn the behavior information in the video session better, inspired by the target detection algorithm, this paper first applies region proposal network to the algorithm to extract the exact region of the person in the video session and transform it to the original image size, which is used as the input of the neural network. Considering the fact that the target area obtained from the image after the target detection algorithm must be of different sizes, an alignment operation is performed on each image to ensure that the images input to the network are of the same size. In addition, this paper performs fragmented sparse sampling of video clips making the model obtain video-level expressiveness, and improves the cross-entropy function for classification to focal loss function to solve the problem of difficult category judgments and possible sample imbalance in classification problems. The main contributions of this paper are as follows:

1. This paper first clearly combs the key deep learning-based methods about behavior recognition, from the classical to the effective.
2. In order to eliminate a large amount of redundant background information in the original video session, we add the target detection mechanism in the process of behavior recognition, so that the weight and bias of CNN cover human behavior information specifically.
3. In order to obtain the time domain information of long-time video, we establish the RGB network of video level representation, so as to conduct the fragmentation and stochastic sampling in the video session.
4. In order to solve the problems of unbalanced sample classification and difficult classification of individual categories, we introduce the improved loss function to recognize human behavior.
5. In order to compare with other classic behavior recognition algorithms, we conducted experiments on UCF-101, HMDB-51 & Kinetics, and the results proved the effectiveness of the proposed method.

## Proposed method

### Target detection

**Fig 2:**
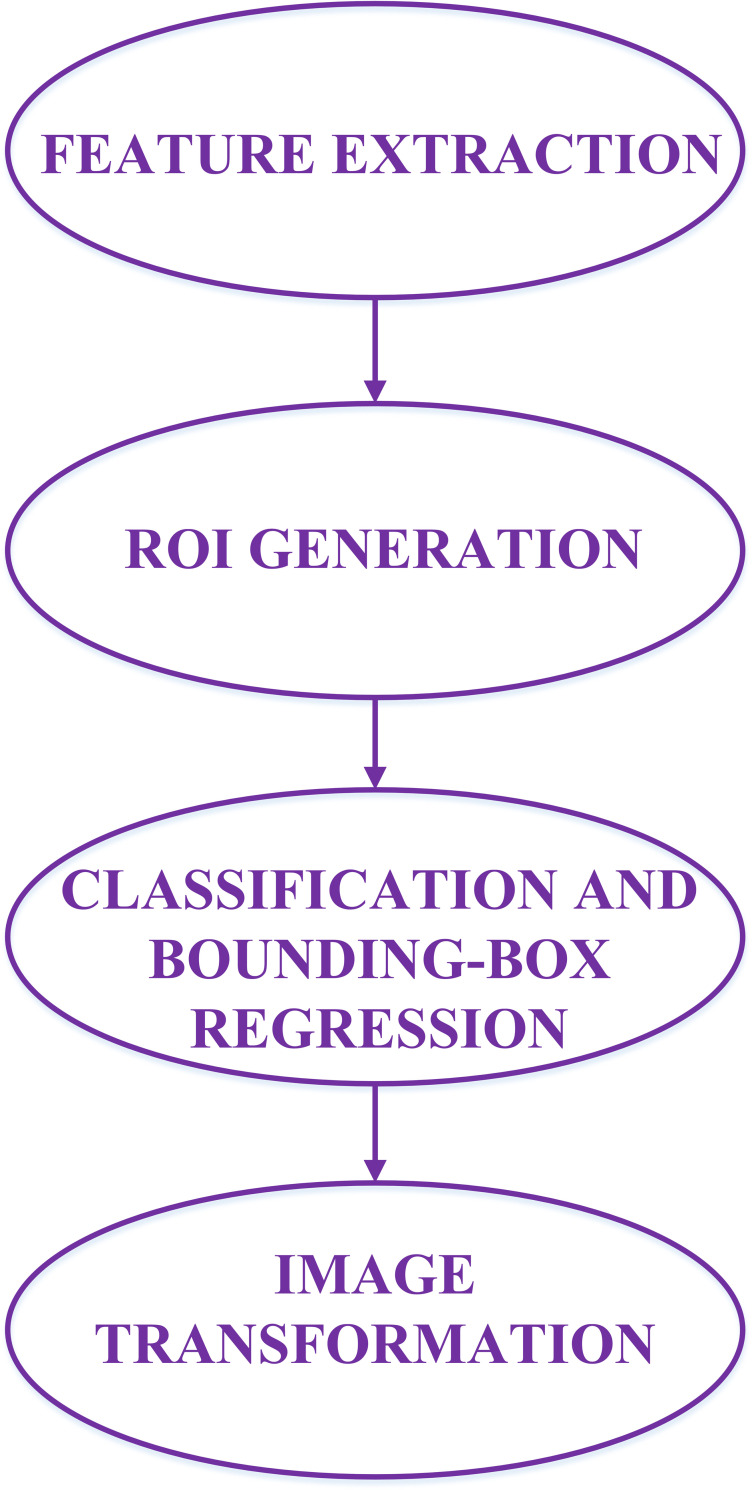
The flowchart about our target detection process.

To combine the accuracy and speed of target detection algorithms, this paper adopts the Faster-RCNN method [27] as the backbone framework for target detection. Firstly, the feature of each image is extracted by a specific feature extraction network, and the obtained feature map is used to generate about 2,000 target candidate regions by the region candidate network. Secondly, the 2,000 target candidate regions are fed to ROI pooling layer for obtaining the region of interest (ROI), and the ROIs pass through the fully connected layer to generate two branches, and then by bounding-box regression and softmax layer, the precise location information of the target region and the probability of the corresponding category are obtained. Finally, the two information from the above target detection algorithm are adjusted to obtain the cropped image and warped image of the target region. The specific process of Faster-RCNN target detection algorithm is as Fig 2 and follows.

### Feature extraction

**Fig 3:**
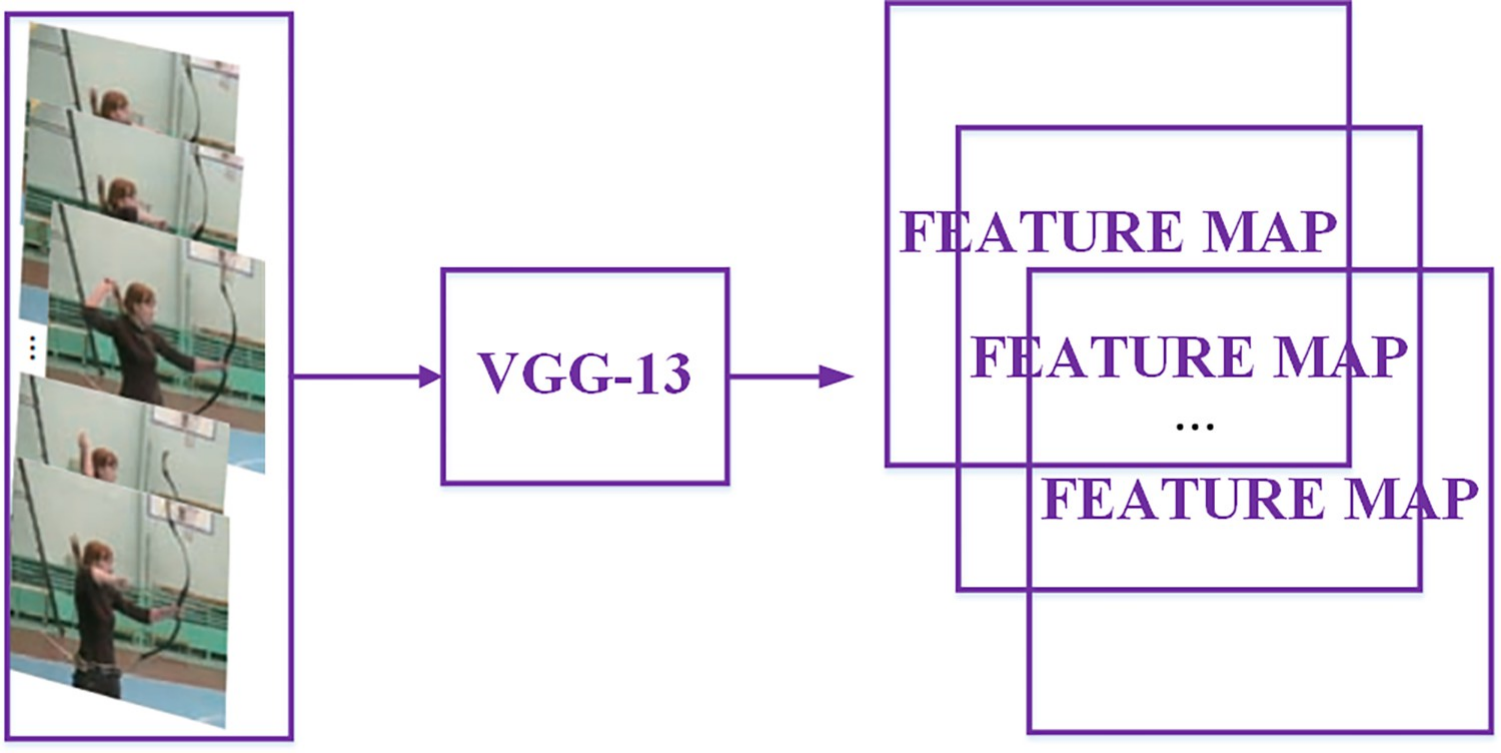
The feature extraction process.

In this paper, we use the pre-trained VGG-13 as the feature extraction network for target detection to extract the feature maps of video frames, and the diagram is shown in Fig 3. VGG-13 has 13 convolutional layers; the size of convolutional kernel is 3 × 3; the zero-padding value is 1; the horizontal and vertical step of convolutional kernel is 1. The relationship among the feature map size (W_1_×H_1_), the original image size (W_0_×H_0_) and the convolutional layer parameters (K×K is the kernel size, *p* is the zero-padding value and *s* is step value) as:

$$W_{1}=\frac{W_{0}-2K+p}{s}+1$$ (1)

$$H_{1}=\frac{H_{0}-2K+p}{s}+1$$ (2)

Each convolutional layer is followed by an activation layer, which does not change the size of the image, so the size of the feature map of the original image after the convolutional and the activation layer does not change. 4 max pooling layers down-sample the output of the activation layer by 2×2 non-overlapping max pooling, so the size of the feature map obtained from the input image through VGG-Net network is 1/16 of the original image size. The final feature map is 512 dimensions, i.e., the feature map size is (W/16) ×(H/16) ×512.

#### The generation of ROI

**Fig 4:**
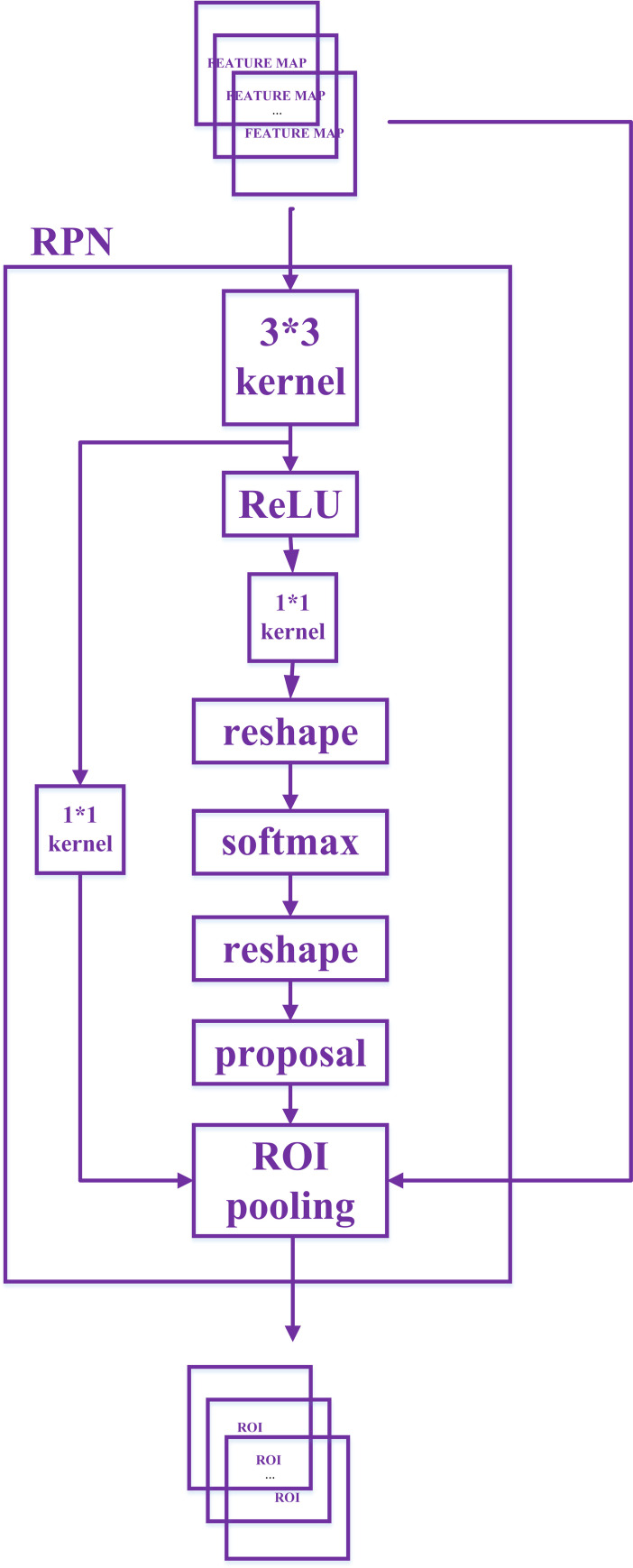
The ROI extraction process.

The region candidate network (RPN) used in this paper is shown in Fig 4. In the RPN, the input feature map goes through convolutional layer that *kernelsize = 3×3*, *padding = 1* and *stride = 1* and activation layer but remains the same size and dimensionality, and then goes through two 1×1 convolutional layers to integrate the information of different dimensions of the feature map and reduce the dimensionality. The anchor output from the middle 1×1 convolutional layer will be used for binary classification to determine whether there is a target in the region, while the anchor output from left 1×1 convolutional layer will be used for bounding-box regression to initially correct the position of bounding-box. The pixel of 1×1 convolutional feature map is mapped to 3 aspect ratios and 3 sizes of regions on the image to generate the anchor. Finally, the ROI is generated from the anchor that may have target information and the preliminarily modified bounding-box information through the ROI pooling layer.

#### Category prediction and bounding-box regression

**Fig 5:**
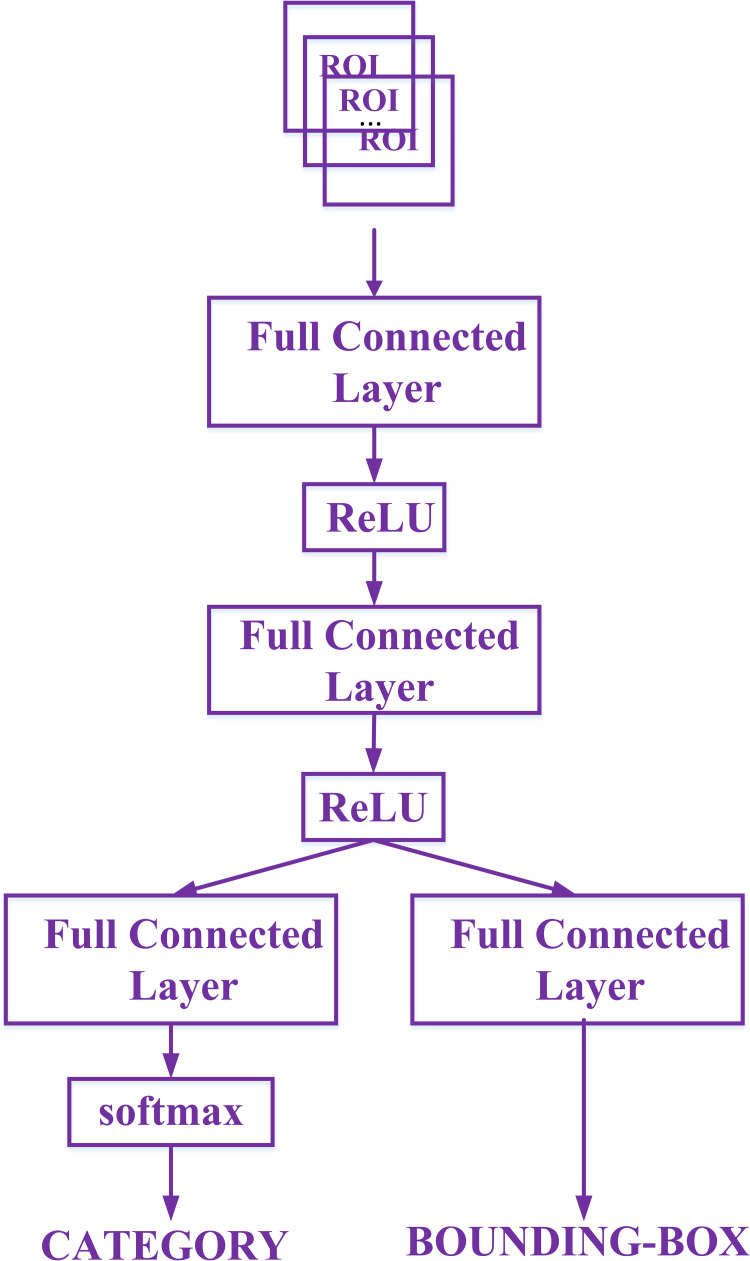
The bounding-box regression and category prediction process.

The generated ROI go through two fully connected layers and the activation layer, and then enter two different fully connected layers for classification and bounding-box regression, final output the probability of ROI belonging to a certain category and the exact bounding-box location information. The flowchart of bounding-box regression and category prediction is shown in Fig 5.

#### Image transformation

**Fig 6:**
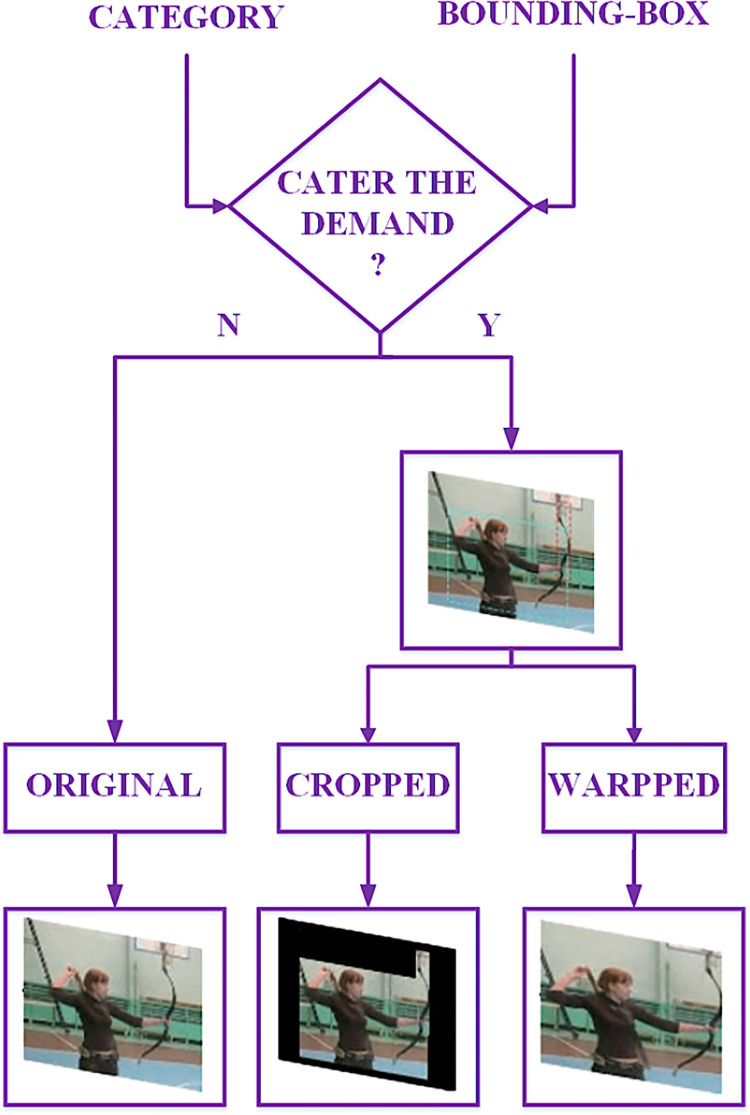
The image transformation process.

Through the target detection algorithm, the bounding-box of the target and the corresponding class can be obtained. Filling the non-target area with black to get the cropped image and expand the target area to the original image size to get the warped image. For the images where no information about the person is found or the images in which total area of all target anchors is less than 1/8 in the original image area, they will be taken as the training sample. The reasons for adopting this method as follows: 1) smaller regions usually contain less image information theoretically; 2) the image in which people not be detected may lose the subject information. Considering the uncertainty of the size of the person target in the image and in order to extract the necessary contextual information in the image, this paper expands the target region to 80 classes in the COCO dataset, forming a target region extraction with the person as the main subject. The flowchart is shown in Fig 6.

### Video segment random sampling and training

#### Video fragmenting and sampling

**Fig 7:**
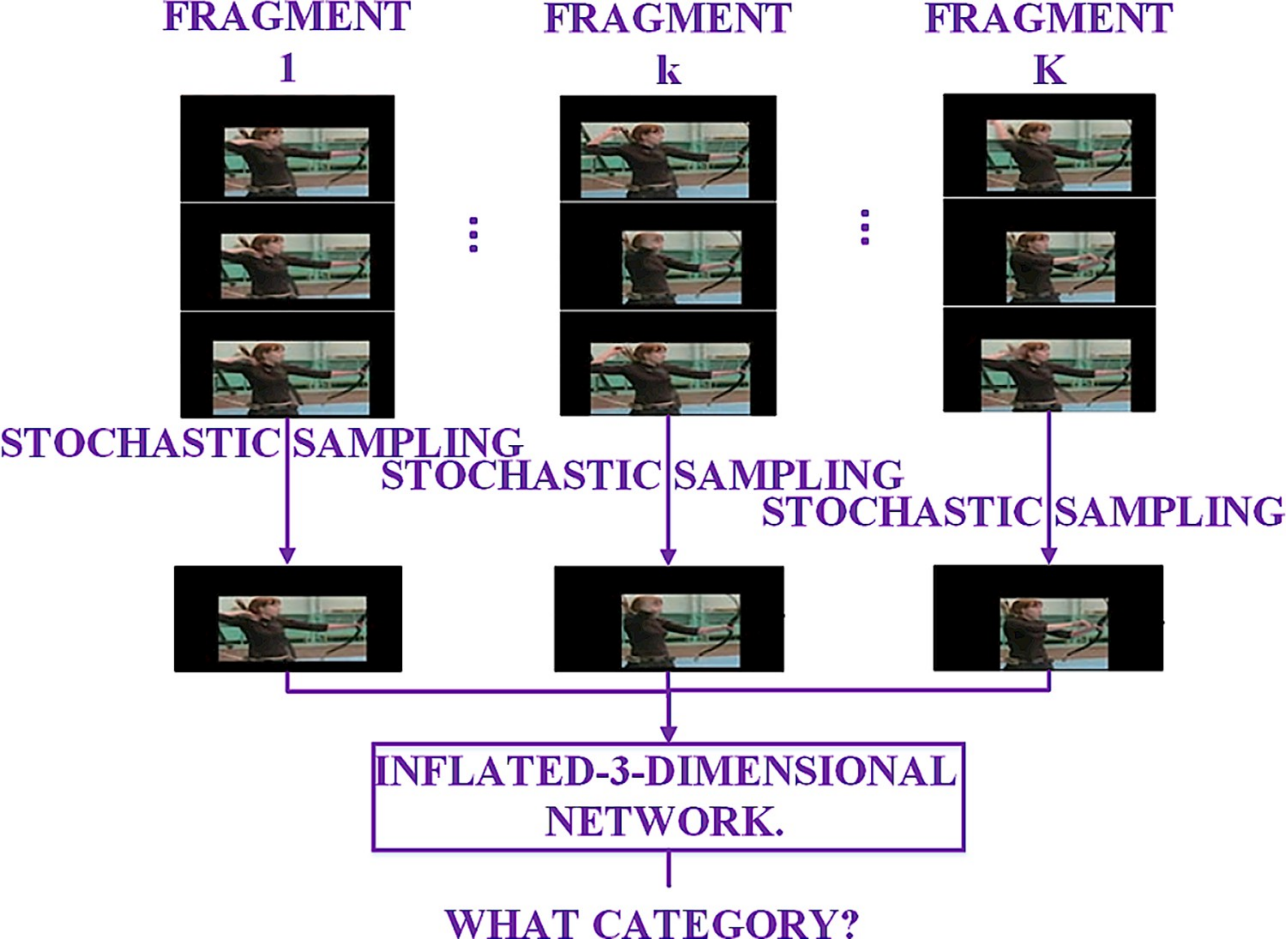
The process of fragment and stochastic sample about video.

In order to obtain the long-time time-domain information of the video, we build the RGB network with video-level representation, as shown in Fig 7, and the video frames are fragmented and stochastically sampled during the training phase. The reasons for using video fragmentation stochastic sampling are: 1) there is a large amount of redundant information in the stacked continuous video frames, and 2) many methods are based on local inference and thus lose the correlation between behaviors that last seconds or even minutes. This paper proposes a method, similar to [28], which also divides video frames into K fragments, but unlike [28], which randomly samples a snippet from its corresponding fragment, and the category scores of different fragments are fused using fragmental consensus function to generate fragmental consensus, and then the predictions of all patterns are fused to produce the final prediction result. In this paper, N/K frames are captured for each fragment, and the N frames are stacked in temporal order and fed into a pre-trained Inflated-3-Dimensional network for recognition, instead of that each video corresponds to one model, and then model fusion is performed.

#### Inflated-3-dimensional network

**Fig 8:**
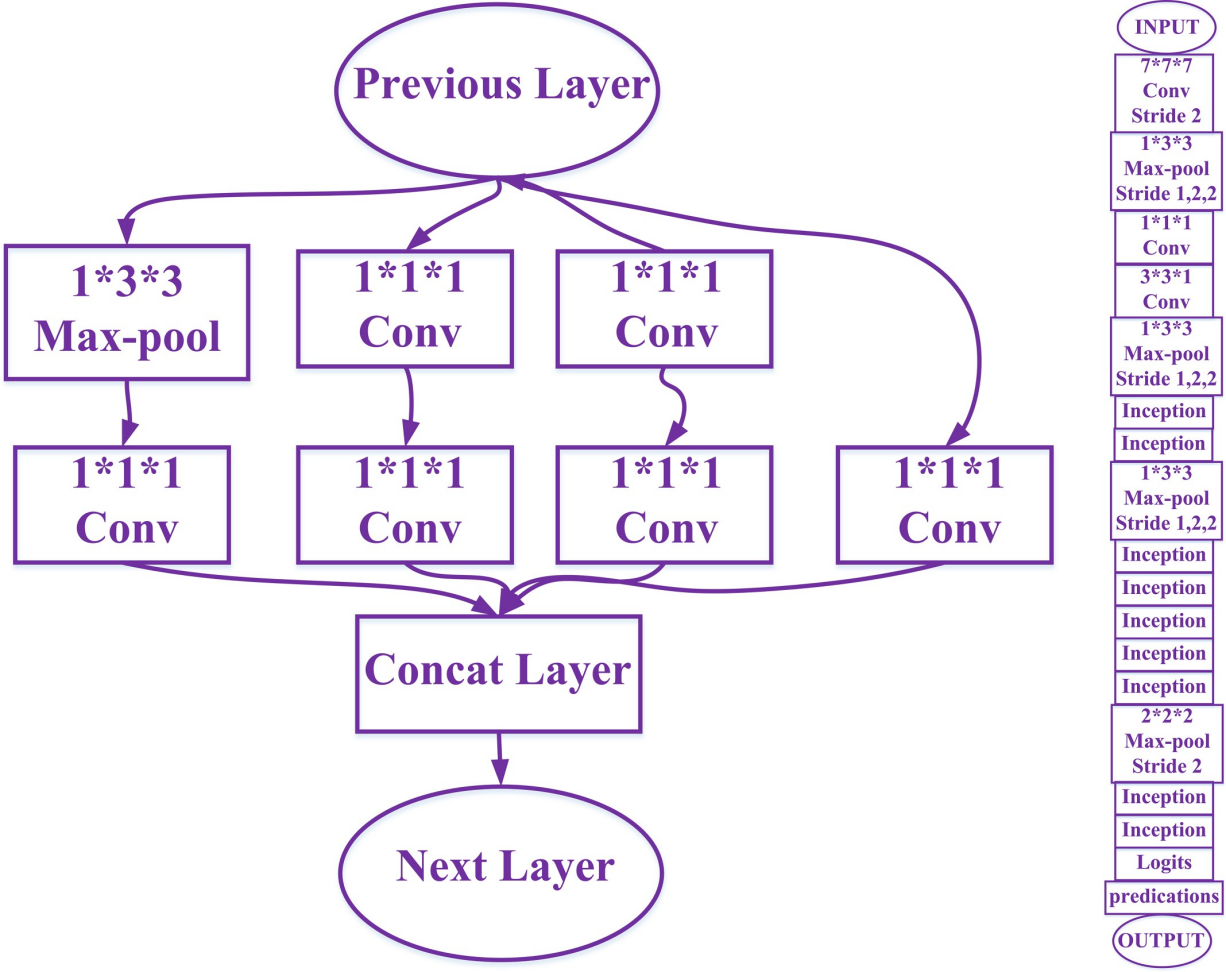
The left side is inflated-3-dimensional network, and the right side is the inception structure.

The implementation of Inflated-3-Dimensional network extends Inception-v1 from 2-dimension to 3-dimension. For a 2-dimensional model, a time dimension is added to all its filters and pooling kernels, e.g., turning N × N filter into N × N × N filter, obtaining 3-dimensional filters from 2-dimensional filters. Replicate the N × N filter N times and then divide it by N for normalization to determine the size of the receptive field in space, time and network depth. The 2-dimensional network and the corresponding 3-dimensional network keep the same kernel size and step size in horizontal and vertical directions, while the 3-dimensional network has a free kernel size and step size in the temporal dimension, which will merge the edge information of different objects if the receptive field size in the temporal dimension is larger than that in the spatial dimension. Otherwise, the dynamic scene will not be captured, and the structure of Inflated-3-Dimensional network is shown in Fig 8 [29].

#### Loss function

The traditional final loss function in the Inception framework is the normal cross-entropy function as (3) & (4), with *p* and *y* being the predicted value and the ground-truth label, respectively:

$$CE(p,y)=\{\begin{array}{l} -\ln(p),\;if(y=1)\\ -\ln(1-p),\;otherwise \end{array}$$ (3)

And rewrite it as:

$$CE(p,y)=CE(p_{t})=-ln(p_{t})$$ (4)

$$s.t.\;p_{t}=\{\begin{array}{l} p,\;if(y=1)\\ 1-p,\;otherwise \end{array}$$ (5)

In this paper, we replace it with focal loss function to deal with the problem of difficult sample classification, and the converted loss function as:

$$Focal\_Loss(p_{t})=-\alpha {\left(1-p_{t}\right)}^{\gamma }ln(p_{t})$$ (6)

Focal loss function can be formed by combining (7) and (8),

$$CE(p_{t})=-\alpha \ln(p_{t})$$ (7)

$$CE(p_{t})=-{\left(1-p_{t}\right)}^{\gamma }log(p_{t})$$ (8)

where (7) adds a modulation parameter α to the cross-entropy, and the values of α is: when y = 1, α = a; and y = −1, α = 1−a. When the proportion of positive samples is much smaller than that of negative samples, a = 0.5~1 is used to increase the weight of positive samples on the total loss function. This solves the positive and negative class imbalance problem. The modulation parameter γ is introduced in (8). If a sample is misclassified, γ tends to 1 when *p*_*t*_ tends to 0, which means that the loss is essentially unchanged compared to the original loss without the modulation parameter; when *p*_*t*_ tends to 1, and the sample is correctly classified and easy to be classified, then γ tends to 0, which means that this type of loss has little weight in the total loss.

## Experiment

### The dataset

To illustrate the effectiveness, we evaluate the proposed method and the current mainstream methods based on the common behavior recognition datasets, mainly the popular scene-related datasets, i.e., UCF-101, HMDB-51 and Kinetics [29], in which the spatial cues, such as scenes, objects and background are dominant for behavior recognition.

#### UCF-101

UCF-101 dataset is an action recognition dataset of realistic behavior videos with 101 behavior categories collected from YOUTUBE, and it is an extension of the UCF50 dataset. With 13,320 videos from 101 behavior categories, UCF-101 offers the greatest diversity in the human behavior, and with large variations in camera motion, target appearance and pose, target scale, angle of view, background, lighting condition and etc. It is still a challenging dataset to date. The videos in the 101 behavior categories are divided into 25 groups, and each group contains 4~7 action videos. Videos from the same group may share some common features, such as similar backgrounds, similar views and etc. The typical behavior categories can be classified into 5 types: 1) human-target interaction; 2) body movement only; 3) human-human interaction; 4) playing a musical instrument; 5) movement.

#### HMDB-51

The content of HMDB-51 dataset is mainly from movies, with a small portion from public databases such as the PRELINGER archive, YOUTUBE and GOOGLE video. The dataset contains 6,849 clips, divided into 51 behavior categories, and each category containing at least 101 clips. The typical behavior categories can be divided into 5 types: 1) general facial behavior; 2) target manipulated facial behavior; 3) general body movement; 4) body movement with target interaction; 5) body movement with human interaction.

#### Kinetics

Kinetics is the largest unconstrained motion recognition dataset to date, containing approximately 300,000 video clips retrieved from YouTube covering up to 400 human actions, ranging from everyday activities and sports scenes to complex interactive actions, with each clip in the video lasting about 10 seconds.

### The experiment setting

The experimental computer configuration is Intel Core i5-8500@ 3.0 GHz with NVIDA GeForce 1080 TI GPU, and the operating system is Windows 10. In the experiments, CNN is designed and implemented based on the Tensorflow platform. In order to prevent over-fitting and build sufficient training feature set, we followed [30] for data augmentation. A spatial position from four corners and the center and a scale from {1,1/2^1/4^,1/2^1/2^,1/2^3/4^,1/2),} were randomly selected for each input sequence for multi-scale cropping. Subsequently, the resolution of the image was uniformly adjusted to 112*112. We also flipped all the frames in the input sequence with a probability of 50%, and also performed mean subtraction and normalization. The network is trained by a mini-batch gradient descent method with a momentum of 0.9, the initial learning rate is set to 0.001 and weights decaying once every 10 epochs with a decay rate of 0.1. We update a total of 60 epochs when training from pre-trained weights in UCF101 and HMDB-51 and that of 160 epochs when training from scratch in Kinetics. All the trainable weights are initialized with orthogonal initializer. The batch size is 6 for HMDB-51 and UCF-101 and 32 for Kinetics.

### The determination of hyperparameters

**Fig 9:**
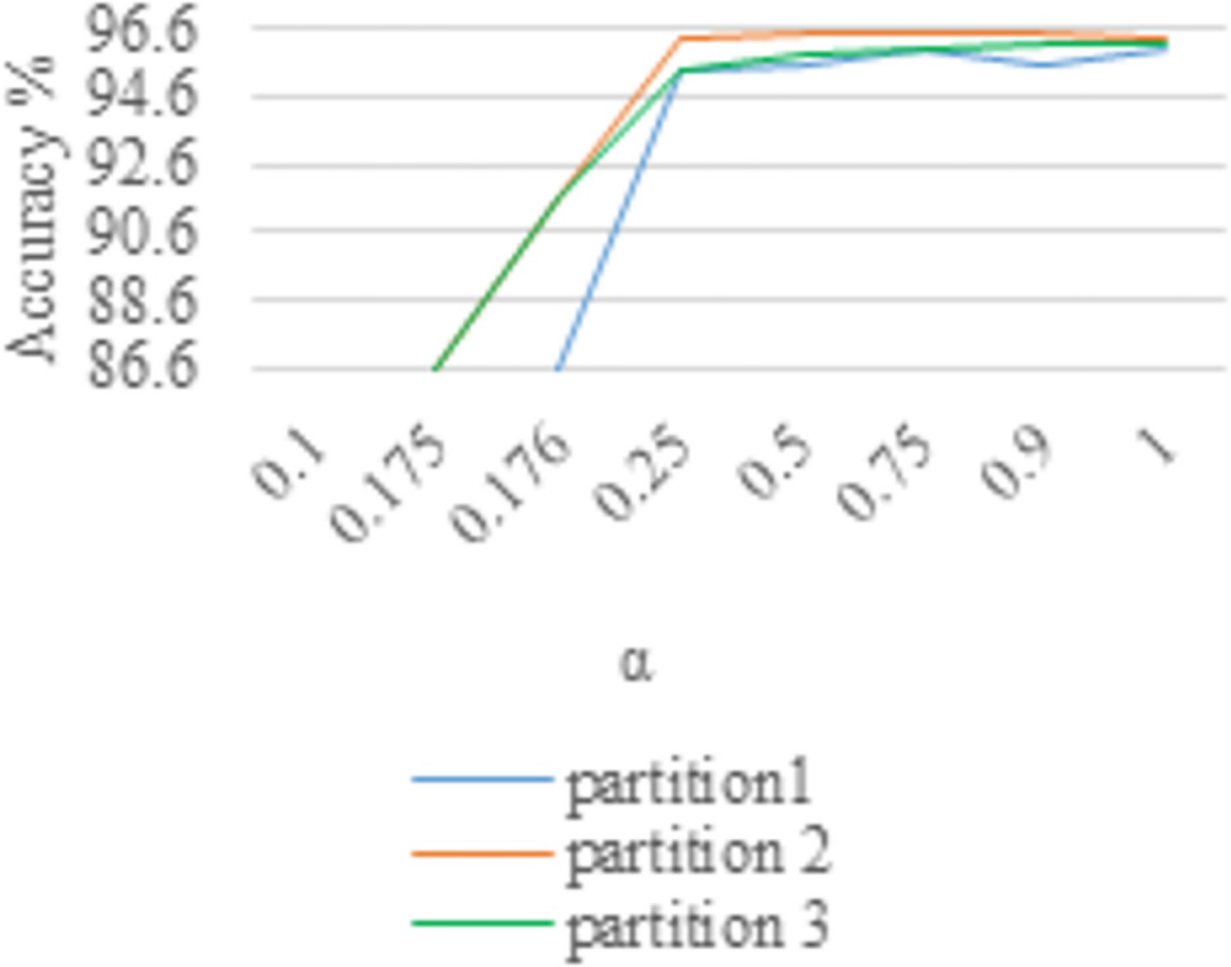
The influence of different values of α on UCF-101 about accuracy.

**Fig 10:**
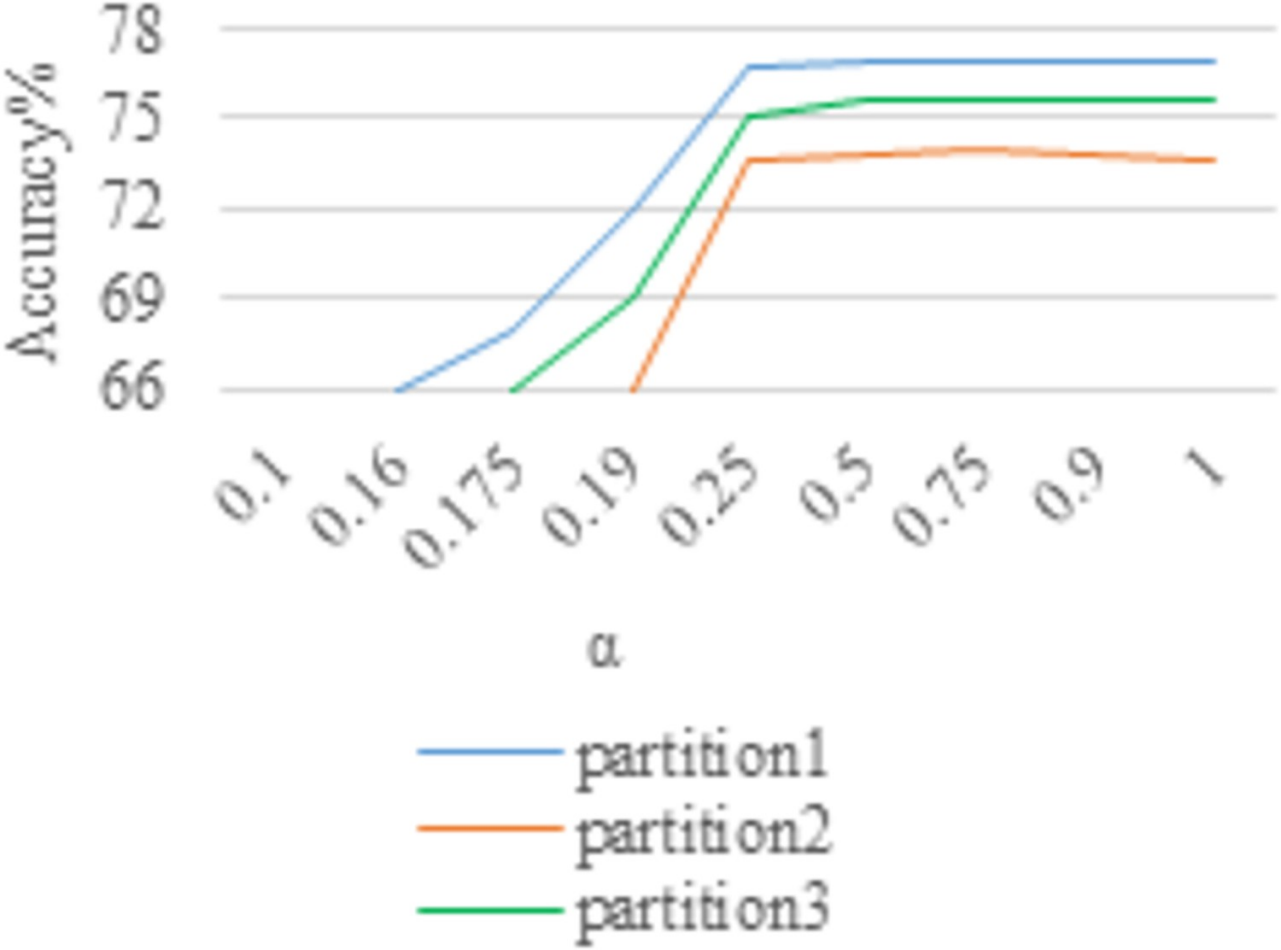
The influence of different values of α on HMDB-51 about accuracy.

Both Figs 9 and 10 show the sensitivity curves of the focal loss parameter α. Both Tables 1 and 2 show the influence of different partitions and the value of α on the accuracy of the two data sets. Note that the inputs are warped image and cropped image, with video fragmented stochastic sampling. From the experimental results, the parameter α of focal loss function has little effect on the experimental results of both datasets after a certain point (i.e., 0.25). However, α = 0.5 and α = 0.75 are slightly improved over the other values for HMDB-51 and UCF-101 datasets, respectively. Because when the parameter α is large (i.e., greater than 0.25), the accuracy in HMDB-51 and UCF-101 datasets is not sensitive to it (from Figs 9 and 10, Tables 1 and 2).

**Table 1:** The effect of different α and different partitions on hmdb-51 about accuracy.

*α*	Partition1	Partition2	Partition3	Average
0.10	60.6	56.5	58.7	58.6
0.25	76.6	73.6	74.9	75.0
0.50	***76*.*8***	73.8	***75*.*2***	***75*.*3***
0.75	76.7	***73*.*9***	75.1	75.2
0.90	76.7	73.8	75.1	75.2
1.00	76.7	73.8	75.1	75.2

**Table 2:** The effect of different α and different partitions on ucf-101 about accuracy.

*α*	Partition1	Partition2	Partition3	Average
0.1	76.8	77.4	78.4	77.5
0.25	95.4	96.3	95.4	95.7
0.5	95.5	96.3	***95*.*9***	***95*.*9***
0.75	***95*.*7***	***96*.*4***	95.6	***95*.*9***
0.9	95.5	96.2	95.7	95.8
1	95.6	96.3	95.8	***95*.*9***

In view of this, we set γ from small to large for the experiments. Both Tables 3 and 4 show the variation of the experimental accuracy with parameter γ. Summing up the above, two parameters α and γ in the focal loss function are coordinated to control each other. Specifically, the parameters used in this paper are α = 0.5 and γ = 5 for the experiment on HMDB-51 dataset and α = 0.75 and γ = 5 for the experiment on UCF-101 dataset.

**Table 3:** The effect of different combinations of α and γ on hmdb-51 about accuracy.

	Partition1	Partition2	Partition3	Average
*α* = 0.50 & *γ* = 0.50	65.3	62.8	63.5	63.9
*α* = 0.5 & *γ* = 0.75	70.8	67.5	69.2	69.2
*α* = 0.50 & *γ* = 2.00	76.6	73.7	75.1	75.1
*α* = 0.50 & *γ* = 5.00	***76*.*9***	***73*.*8***	***75*.*3***	***75*.*3***
*α* = 0.75 & *γ* = 3.00	76.7	73.7	75.2	75.2
*α* = 0.75 & *γ* = 5.00	76.7	73.7	75.1	75.2
*α* = 0.90 & *γ* = 10.0	76.3	73.4	74.7	74.8

**Table 4:** The effect of different combinations of α and γ on ucf-101 about accuracy.

	Partition1	Partition2	Partition3	Average
*α* = 0.50 & *γ* = 0.50	78.3	78.9	77.4	78.2
*α* = 0.50 & *γ* = 0.75	86.8	88.4	87.4	87.5
*α* = 0.50 & *γ* = 2.00	95.4	96.3	***96***	95.9
*α* = 0.50 & *γ* = 5.00	95.6	96.3	95.8	95.9
*α* = 0.75 & *γ* = 3.00	95.5	96.2	95.7	95.8
*α* = 0.75 & *γ* = 5.00	***95*.*7***	***96*.*4***	95.9	***96***
*α* = 0.90 & *γ* = 10.0	95	95.9	95.5	95.5

### The ablation experiment

Both Tables 5 and 6 show the recognition results of proposed algorithm on the behavior recognition datasets UCF-101 and HMDB-51 using different input images, warped images and cropped images. The dataset is portioned as three partitions for training and testing the accuracy, and finally averaging the results of all the test sets.

**Table 5:** The effect of different combinations of input on hmdb-51 about accuracy.

Input	Partition1	Partition2	Partition3	Average
CROP.	87.6	91.7	90.9	90.1
WROP.	90.4	92.2	92.5	91.7
ORI.	95.2	95.8	95.4	95.5
CROP. + ORI.	91.7	92.7	92.9	92.4
WROP. + ORI.	***95*.*7***	***96*.*4***	***96*.*0***	***96*.*0***

**Table 6:** The effect of different combinations of input ucf-101 about accuracy.

Input	Partition1	Partition2	Partition3	Average
CROP.	71.3	67.1	68.8	69.7
WROP.	74.1	70.2	70.6	71.6
ORI.	75.9	73.1	75.0	74.7
CROP. + ORI.	73.3	71.8	72.0	72.4
WROP. + ORI.	***76*.*8***	***73*.*9***	***75*.*3***	**75.3**

The experimental results show that the warped images are more distinguishable than the cropped images, because the cropped images have more black areas than the warped images. In fact, there are such black areas in each class of images due to the variable anchor size and scale. Therefore, the warped images with the same resolution have less redundant information and more valid information than the cropped images. The warped & original images reduce the noise and increase the proportion of human behavior areas in the image compared with the pure original images, making the training result more efficient.

**Fig 11:**
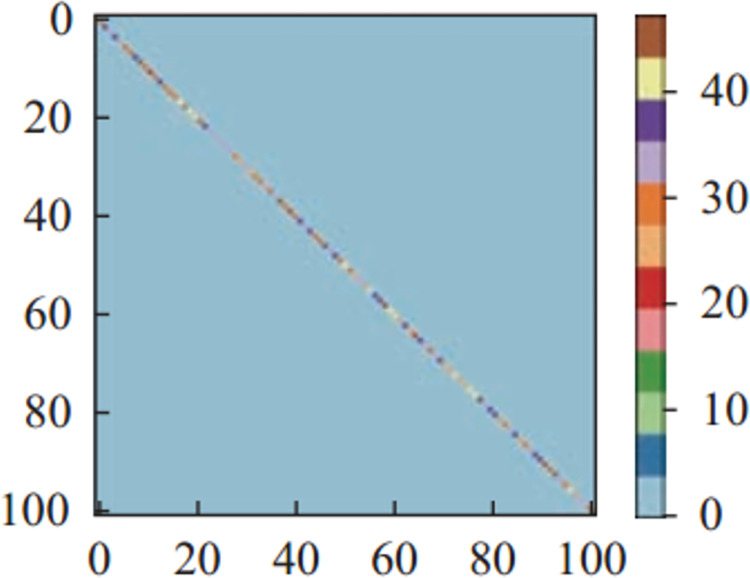
The confusion matrix of the first sectionalization in UCF-101 under the input warped & original image.

**Fig 12:**
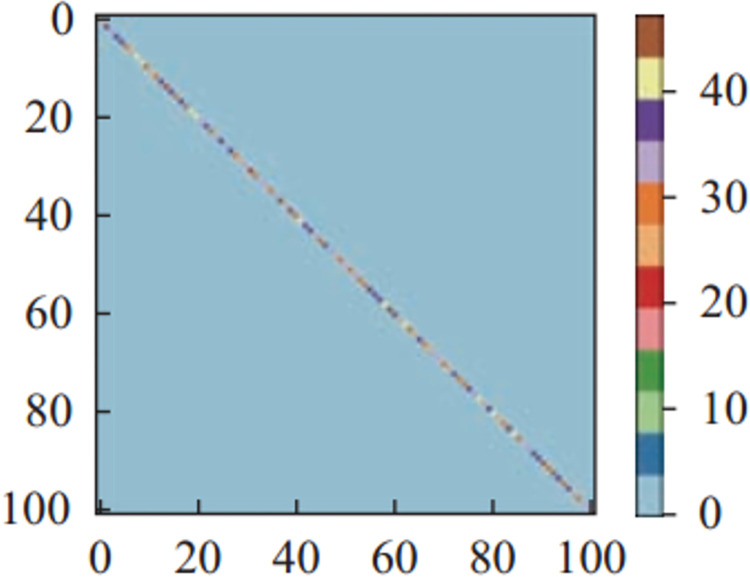
The confusion matrix of the first sectionalization in UCF-101 under the input cropped & original image.

**Fig 13:**
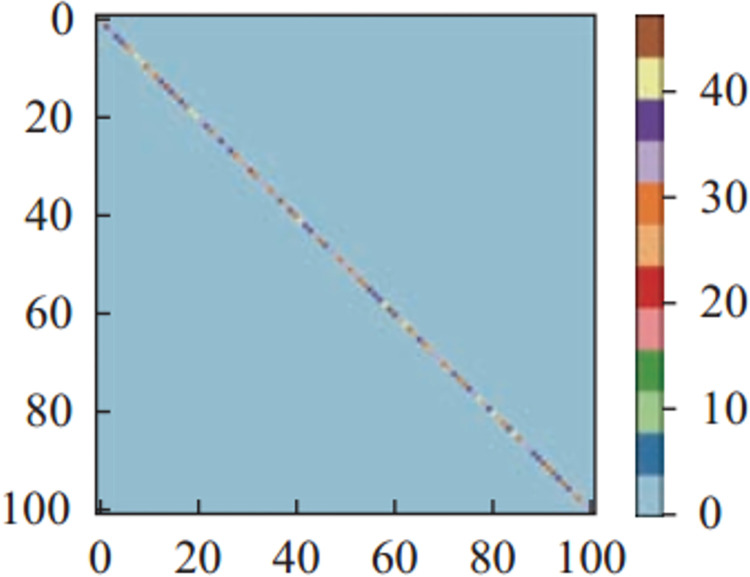
The confusion matrix of the first sectionalization in UCF-101 under the input cropped image.

**Fig 14:**
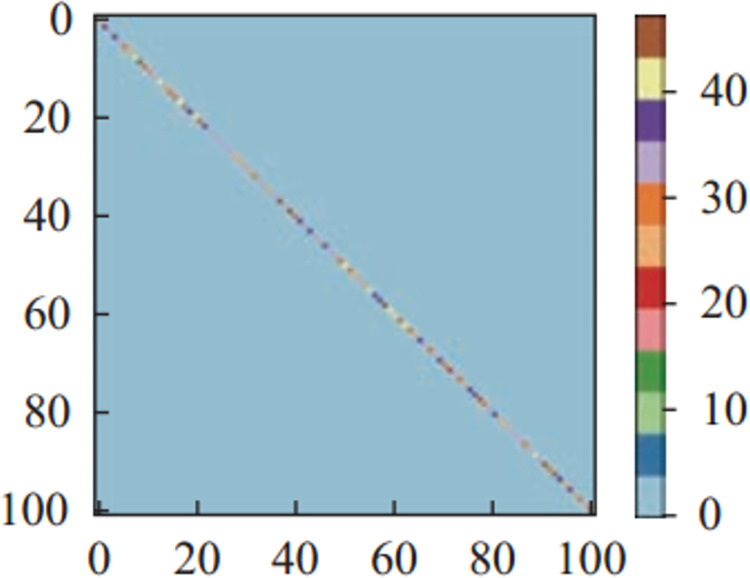
The confusion matrix of the first sectionalization in HMDB-51 under the input warped & original image.

**Fig 15:**
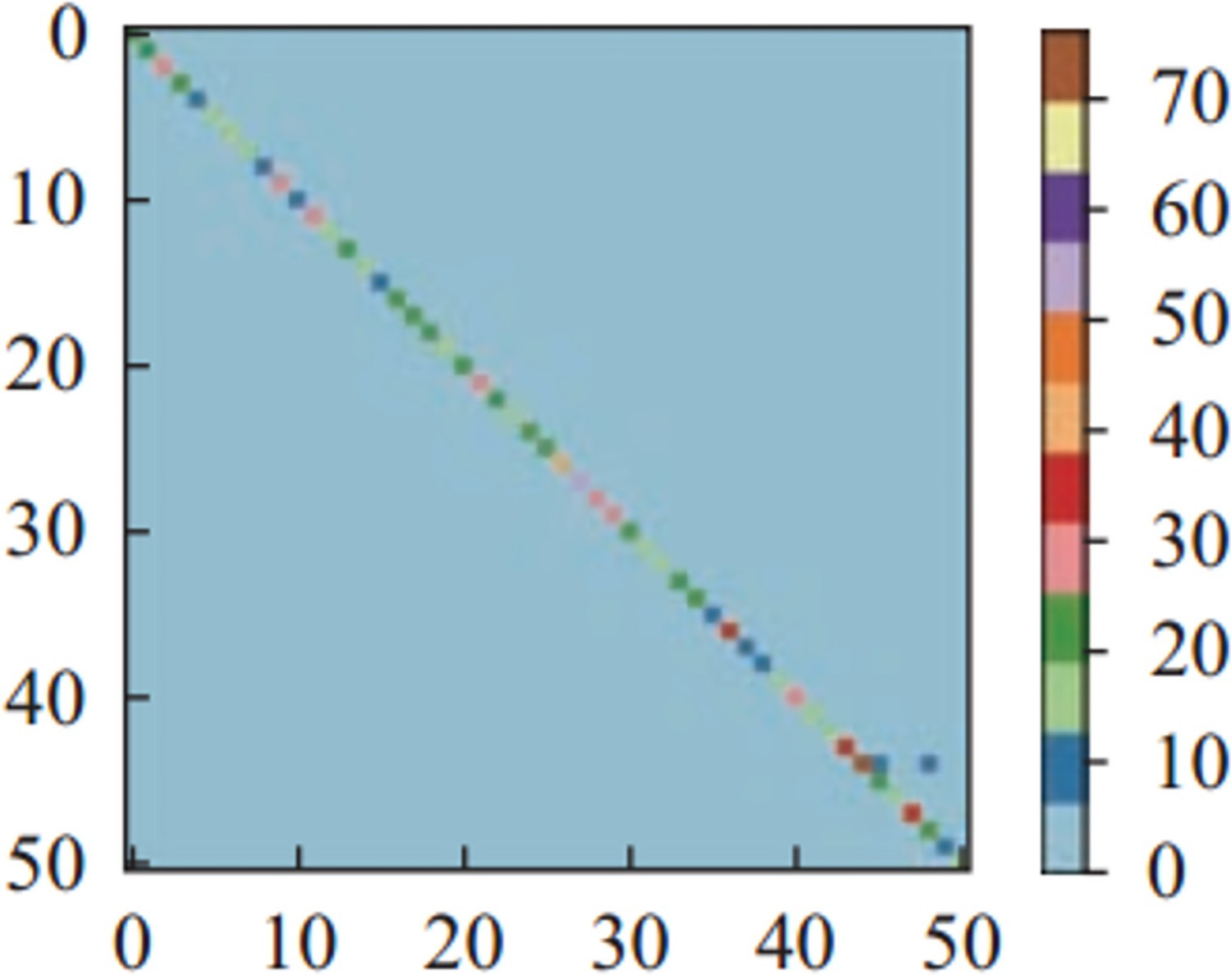
The confusion matrix of the first sectionalization in HMDB-51 under the input cropped & original image.

**Fig 16:**
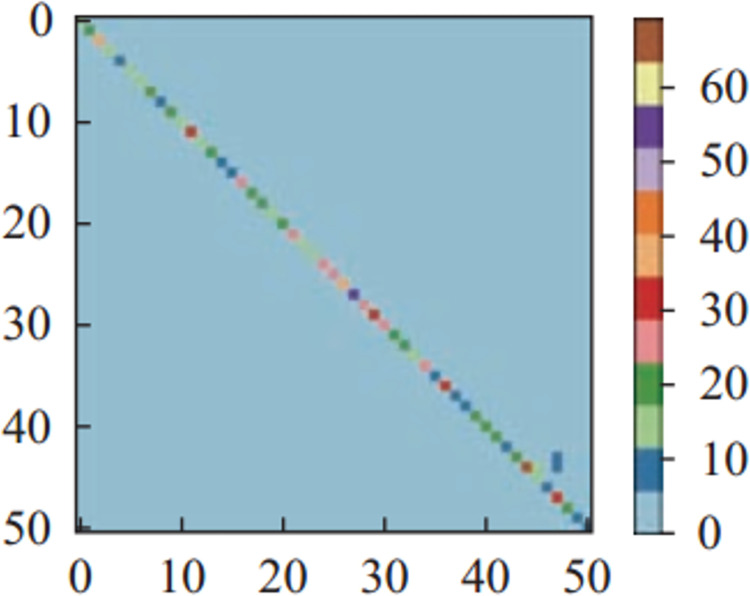
The confusion matrix of the first sectionalization in HMDB-51 under the input cropped image.

Figs 11–13 and 14–16 show the confusion matrices in UCF-101 and HMDB-51 datasets respectively for different categories of images on the test set of the first sectionalization. UCF-101 dataset cannot visualize the difference in the accuracy of the model when predicting each category from the confusion matrix due to the high prediction accuracy, while HMDB-51 dataset clearly shows that the prediction probabilities of the warped & original images in category-48 and category-49 respectively are significantly higher than the others.

**Fig 17:**
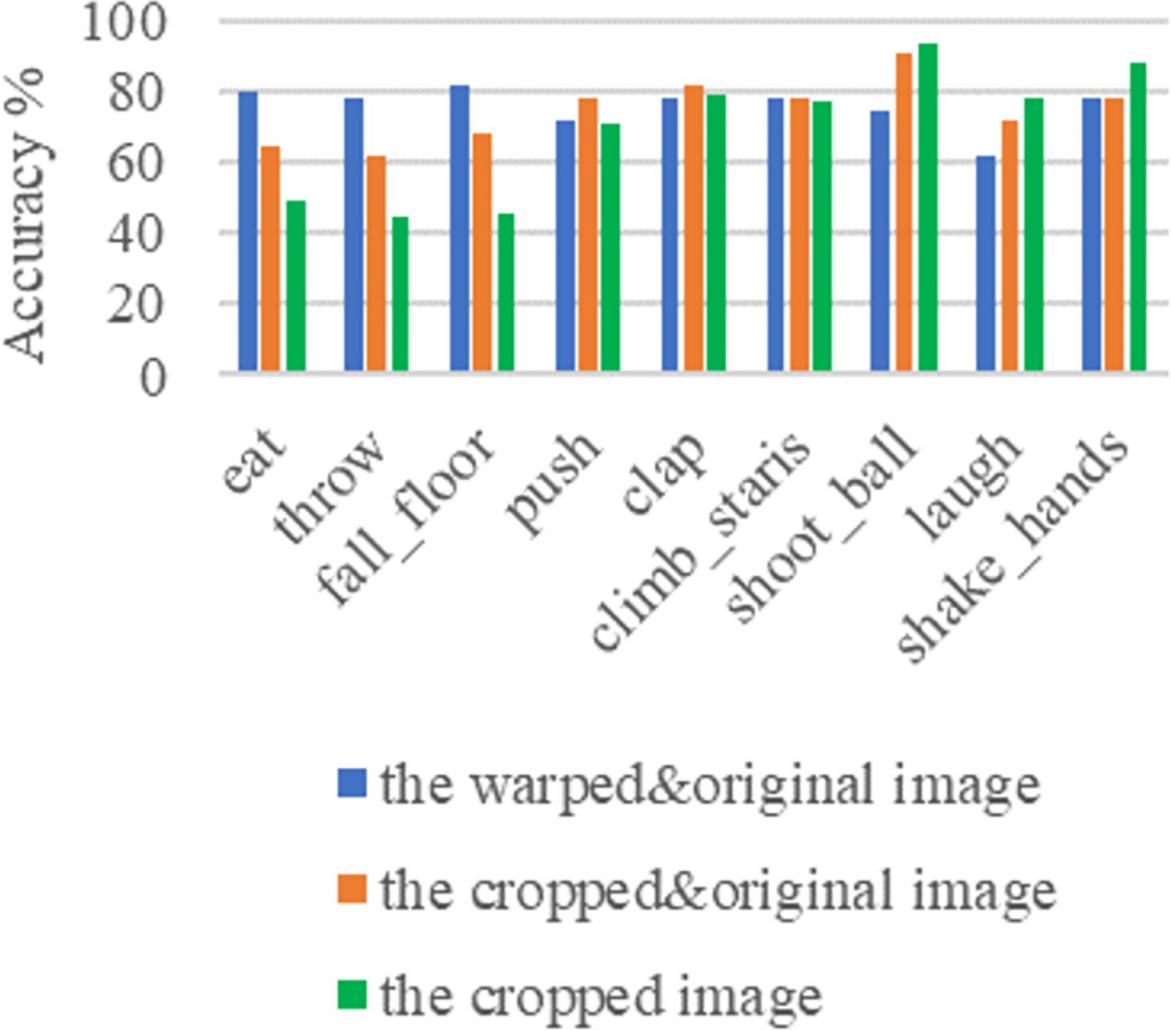
Some typical class accuracy display under different inputs on HMDB-51.

**Fig 18:**
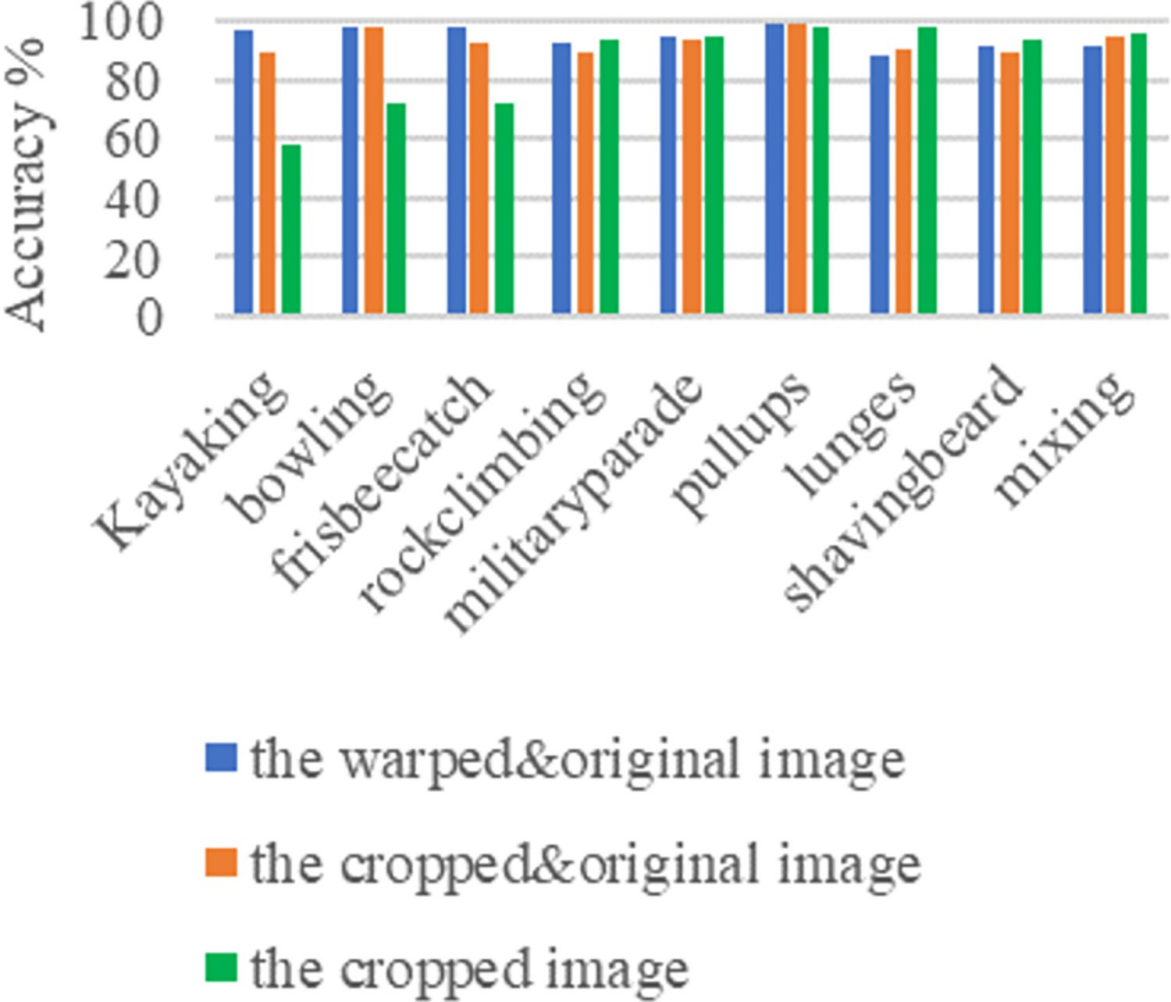
Some typical class accuracy display under different inputs on UCF-101.

Both Figs 17 and 18 show the comparison of the test accuracy of some classes in the Inflated-3-Dimensional network with different input images. The categories with the largest improvement, the smoothest change and the largest decrease about the predicted probability of the warped & original images over the cropped & original images and the original images for both datasets are included. The category-48 *throw* of HMDB-51 located on the most elevated categories, which is consistent with the confusion matrix in Fig 16. The categories with the largest relative increase in both datasets are *eat*, *throw*, *fall_floor*, *kayaking*, *bowling* and *frisbeecatch*. These behavior’s background occupies a larger area relative to the behaviors on the others and are highly correlated with the behaviors. The behaviors with the highest relative decrease are *shoot_ball*, *laugh*, *shake_hands*, *lunges*, *shavingbeard* and *mixing*. These behaviors occupy a small area of the image relative to the human body or have a little movement, so removing the background completely is more effective in improving the recognition rate of this behavior.

### The contrast experiment

Table 7 shows the comparison results of the proposed algorithm with other existing algorithms on the behavior recognition datasets UCF-101 and HMDB-51. The following is a brief introduction of the contrast methods. Yan et al. [31] learned video representations using neural networks with long-term temporal convolutions (LTC), Furtherly, they demonstrated that LTC-CNN models with increased temporal extents improve the accuracy of behavior recognition. Al et al. [32] built upon two-stream CNN and proposed deep networks with temporal pyramid pooling, an end-to-end video-level representation learning approach, to address behavior recognition problem. Le et al. [33] presented an empirical CNN architecture search for spatiotemporal feature learning, culminating in a deep 3-dimensional residual CNN. Bobick et al. [34] introduced a new temporal layer that model variable temporal convolution kernel depths, and embed this new temporal layer in the proposed 3-dimensional CNN. Liang et al. [35] presented a new architecture, termed as appearance-and-relation network to learn video representation in an end-to-end manner. Their network was constructed by stacking multiple generic building blocks, whose goal is to simultaneously model appearance and relation from RGB input in a separate and explicit manner. Hu et al. [36] introduced a new dual-stream inflated 3-dimensional CNN that is based on 2-dimensional CNN inflation: filters and pooling kernels of very deep image classification CNN are expanded into 3-dimension, making it possible to learn seamless spatial-temporal feature extractors from video while leveraging successful ImageNet architecture designs and even their parameters. Laptev et al. [30] used a single RGB image as the input of a deep learning model, which only considered the spatial apparent features of the video and ignored the difference between the video and a single still image, without encoding the temporal information of the video. In this regard, Niebles et al. [37] first used 3-dimensional-CNN to obtain motion information. Song et al. [26] used 2- dimensional-CNN to extract the representational information of video frames, followed by connecting a long short-term memory recurrent neural network or gated recurrent unit and etc., which aims to learn the motion information between frames. As the pre-training datasets of some comparison methods are inconsistent with those used by us, they are also marked in Table 7. Specifically, the underlined method has a pretraining dataset of SPORTS-1M, the italicized method has a pretraining dataset of IMAGENET, the bold method has a pretraining dataset of KINETICS, and the method with shading has a pretraining dataset of IMAGENET & KINETICS. From Table 7, without fragmented stochastic sampling and focal loss function, the experimental results show that the target detection algorithm can help effectively learn and discriminate the behavior information of the person in the video session. In this paper, the warped & original image input form is used to balance the deletion of excessive background information with the retention of necessary background information, which can effectively improve the accuracy of behavior recognition. The experiment results show that focal loss function and the video fragmented stochastic sampling strategy further improve the competitiveness of the algorithm in this paper. It also shows the effectiveness of the proposed method in temporal and spatial significance modeling and temporal feature modeling.

**Table 7:** Accuracy comparison of different methods in UCF-101 and HMDB-51.

method	UCF-101	HMDB-51
Yan [31]	82.4	48.7
Al [32]	85.8	54.9
*Du [33]*	*86*.*4*	*53*.*7*
*Diba [34]*	*89*.*7*	*61*.*1*
**Liang [35]**	**89.8**	**62.1**
**Carreira [29]**	**91.7**	**61.1**
**Karpathy [14]**	**94.3**	**70.9**
Ji[43]	91.1	60.5
Donahue[26]	95.6	74.8
OURSwithoutF.S.S.&F.L.	95.7	75.1
OURSwithoutF.L.	95.8	75.1
OURSwithoutF.S.S.	95.8	75.2
OURS(all)	96.1	75.4

To further verify the effectiveness of the proposed method, we also conducted a contrast experiment in Kinetics dataset [10], and the results are shown in Table 8. It is worth noting that Kinetics is often used as a pre-training data set for behavior recognition, so both the proposed method and the contrast methods are based on training from scratch on that data set rather than fine-tuning. The following is a brief introduction of the contrast methods. In the framework of residual learning, Du et al. [38] used 3-dimensional-CNN and decomposed the 3-dimensional convolution filter into separate spatial and rhythmic components, and proposed a new design of spatio-temporal convolution block "R (2+1) D". Diba et al. [39] proposed Spatio-Temporal Channel Correlation (STC) in order to strengthen the Correlation between time domain and space domain of 3-dimensional-CNN. In addition, they also proposed a method that can migrate the pre-trained 2-dimensional-CNN model to 3-dimensional-CNN model, effectively preventing over-fitting. Tran et al. [40] used 3-dimensional group convolution networks and decomposed 3-dimensional convolution by separating channel interactions and space-time interactions to improve accuracy and reduce computational cost. Feichtenhofer et al. [41] proposed a slow video recognition network, which consists of slow paths running at low frame rates to capture spatial semantics, and fast paths running at high frame rates to capture motion with fine temporal resolution. Feichtenhofer [42] introduced X3D, which uses a simple step-by-step network extension approach along multiple network axes of space, time, width and depth, extending a single axis in each step, gradually expanding a tiny 2-dimensional image classification system. Kumawat et al. [19] proposed a spatio-temporal short-term Fourier transform (STFT) block, which can be used as a substitute for 3-dimensional convolution layer and its variants in 3D CNN. The STFT block consists of untrainable convolution layers that use the STFT kernel to capture local Fourier information spatially and/or spatially at multiple low-frequency points, followed by a set of trainable linear weights for learning channel correlation. It can be seen from the results that the performance of the proposed method in big data sets is still quite excellent, which shows that the proposed method is more reliable in terms of spatio-temporal attention, and proves the good generalization and robustness of the proposed method.

**Table 8:** Accuracy comparison of different methods in kinetics.

method	Top-1	Top-5
Ji [37]	67.5	87.2
Le [38]	72.0	90.0
Bobick [39]	68.7	88.5
Tran [40]	77.8	93.5
Feichtenhofer [41]	78.7	62.1
Feichtenhofer [42]	77.5	92.9
Kumawat [19]	72.2	90.4
OURS without F.S.S. &F.L.	76.5	89.2
OURS without F.L.	78.9	93.4
OURS without F.S.S.	79.1	94.7
OURS (all)	80.7	95.1

## Discussion

As described in the previous section, It can be proved that the target object mechanism of this paper can suppress the temporal and spatial background noise effectively; the fragmentation and stochastic sampling in the video session mechanism of this paper further improves the reliability of the behavior spatio-temporal estimation and the prospect information extraction. Combined with improved loss function, the proposed method can improve the accuracy of behavior recognition in long-time video. However, there are some other problems that can be enough to affect the precision of the recognition:

1. Intra-class differences and inter-class similarities of behaviors. Different people do the same kind of behavior, even if the same person does the same kind of behavior, the result in the video may be very different due to individual differences, movement speed, environment and background. And different kinds of movements may show very similar characteristics. With the increase of the number of behavior categories, the degree of overlap between different gestures increases with the subdivision of the representation space, that is, there is often a large intra-class divergence between similar behaviors, while a small inter-class divergence between different action classes, which is also a challenge to the recognition of behaviors.
2. The annotation of large amount of training data / Small sample learning / Unsupervised learning. The research of human behavior recognition has experienced from simple behavior recognition in restricted scenes to behavior recognition in movies and then to human behavior recognition in scenes of natural life. How to label the collected video data is a problem. The method of manual annotation is time-consuming and labor-consuming, which is obviously not advisable. Therefore, the tool or technology for automatic annotation of video data is urgently needed. Or think about it another way. Most behavior recognition methods based on deep learning require a large number of training samples to make the model converge. However, when behavior recognition is applied to a particular scene, there are often not enough training samples, and the lack of training samples may lead to the over-fitting problem of the deep learning model, which will seriously affect the generalization ability of the model. At present, small sample learning has made some progress. For example, Ji et al. [43] proposed the concept of behavior genome, which decomposed behaviors into the form of spatio-temporal scene graph to capture changes between objects and their relationships, making the mainstream behavior recognition model achieve great performance improvement in small sample learning. In addition, Cao et al. [44] designed a timing correction module, which effectively utilized the time sequence information in video data through timing correction and improved the efficiency of data utilization. In the aspect of small sample learning, the problem of less labeled data in small sample learning can be alleviated from the perspective of multi-modal information utilization, such as adding depth map, skeleton map and other information. At the same time, new video data enhancement technology can be introduced and generative adversarial network can be used to enhance data at the feature level to improve the robustness of the model. Second best, video data is also an excellent material for unsupervised learning because it contains a lot of dynamic structure information. Unsupervised video learning mainly includes time autoencoder methods, such as PredNet [45], PredRNN [46] and PredRNN++ [47], but its performance in mass transfer learning has not been confirmed. Recently, it has been mentioned in [48] that deep video embedding can be trained to maximize the distance between different videos and minimize the distance between the same videos. Such visual representations learned from a large number of behavior videos can significantly improve the accuracy of behavior recognition, but it is often difficult to train powerful video embedded visual features on large data sets. In the future, with the help of the ideas in [49], the corresponding relationship between positive and negative labels between two networks can be used to learn the video representation, so as to realize the supervised transfer across networks.
3. Uncertainty of behavior boundary. For untrimmed videos, a video may contain multiple behaviors, some of which last for a short time, while some of which last for a long time and change in speed quickly. Therefore, it is difficult to accurately locate the boundary of the behavior in time sequence, and the fuzzy boundary of the behavior will affect the accuracy of recognition to a large extent.
4. A more streamlined model. At present, most deep learning models still have the characteristics of multiple parameters and high time complexity, which leads to high memory consumption and slow running speed of algorithms, which cannot meet the requirements of real-time and high efficiency, and cannot be run on mobile devices. At present, in order to ensure efficiency and accuracy at the same time, most lightweight models are based on 2-dimensional-CNN, which is capable of learning spatio-temporal features by deploying sequence feature extraction modules with no parameters or fewer parameters. For example, TSM (Temporal Shift Module) [50] GST (Grouped Spatial-Temporal) [51] etc. Meanwhile, with the successful application of ResNext [52] and ShuffleNet [53] in the field of image classification, the sequential feature extraction module can be designed with the help of the idea of channel grouping convolution or deep separable convolution, so as to effectively reduce network parameters while ensuring spatio-temporal interaction. In addition, knowledge distillation is adopted to transfer the knowledge learned by the network with strong complex learning ability, namely when the input-output mapping relationship is transferred to the network with few parameters and weak learning ability, the model can be compressed indirectly.
5. Other uncertainties. There are often problems such as uneven lighting, background changes and etc. in the behavior data set. In the type of human-object interaction video, there will be the phenomenon of object deformation, and in the type of human-human interaction video, there will also be the phenomenon of target occlusion. The interference of these uncertain factors will seriously affect the performance of the model, which is also an important reason for the failure of behavior recognition to be practical.

## Conclusion

Most of the research methods in the field of behavior recognition extract the relevant features from the original video frames, which introduce more or less redundant background information, thus bringing great noise to the convolutional neural network. In order to solve the problems of background information interference, large amount of redundant information existing in video frames, unbalanced sample classification and difficult classification of individual categories, this paper proposed an effective behavior recognition method in the video session using convolutional neural network. Firstly, the target detection phase is added in the process of human behavior recognition, so that the neural network can learn the behavior information more emphatically. Secondly, fragment stochastic sampling is carried out to build a long-time domain model spanning the entire video fragment. Finally, behavior recognition is carried out by the improved loss function of behavior recognition network. We conducted the hyperparametric experiment, the ablation experiment and the contrast experiment on different open source and benchmark dataset. Compared with other commonly used behavior recognition algorithms, the experimental results verify the effectiveness of the proposed method. In addition, the existing problems analyzed in the discussion section are also the direction of our future research.

## References

[1] Cui H., Guan Y. and Chen H. Rolling Element Fault Diagnosis Based on VMD and Sensitivity MCKD[J], IEEE Access, vol. 9, pp. 120297–120308, 2021, doi: 10.1109/ACCESS.2021.3108972

[2] Zhang ZH., Min F., Chen GS. et al. Tri-Partition State Alphabet-Based Sequential Pattern for Multivariate Time Series. Cogn Comput (2021). 10.1007/s12559-021-09871-4.

[3] Deng W., Zhang X., Zhou Y., et al. An enhanced fast non-dominated solution sorting genetic algorithm for multi-objective problems[J]. Information Sciences, 2020, 585:441–453.

[4] Medel J R, Savakis A. Anomaly Detection in Video Using Predictive Convolutional Long Short-Term Memory Networks[J]. 2016.

[5] Liu J, Shahroudy A, Wang G, et al. Skeleton-Based Online Action Prediction Using Scale Selection Network[J]. IEEE Transactions on Pattern Analysis & Machine Intelligence, 2019:1–1. 10.1109/TPAMI.2019.2898954 30762531

[6] Kantorov V, Laptev I. Efficient feature extraction, encoding and classification for action recognition. 2014.

[7] Sadanand S, Corso J J. Action bank: a high-level representation of activity in video [C]. Proc of IEEE Conference on Computer Vision and Pattern Recognition. Piscataway, NJ: IEEE Press, 2012: 1234–1241.

[8] Ran X., Zhou X., Lei M., et al. A Novel K-Means Clustering Algorithm with a Noise Algorithm for Capturing Urban Hotspots[J]. 2021, 11:11202.

[9] Chen J, Wu J, Konrad J, et al. Semi-Coupled Two-Stream Fusion ConvNets for Action Recognition at Extremely Low Resolutions[C]// 2017 IEEE Winter Conference on Applications of Computer Vision (WACV). IEEE, 2017.

[10] Sun Z, Liu J, Ke Q, et al. Human Action Recognition from Various Data Modalities: A Review[J]. 2020. 10.1109/TPAMI.2022.3183112 35700242

[11] Zhang X, Wang H, Du C, et al. Custom-Molded Offloading Footwear Effectively Prevents Recurrence and Amputation, and Lowers Mortality Rates in High-Risk Diabetic Foot Patients: A Multicenter, Prospective Observational Study. Diabetes Metab Syndr Obes. 2022 Jan 10;15:103–109. doi: 10.2147/DMSO.S341364 35046681 PMC8759996

[12] Cui H., Guan Y., Chen H., et al. A Novel Advancing Signal Processing Method Based on Coupled Multi-Stable Stochastic Resonance for Fault Detection[J]. 2011,11(12):5385.

[13] Diba A, Sharma V, Gool L V. Deep Temporal Linear Encoding Networks[C]// 2017 IEEE Conference on Computer Vision and Pattern Recognition (CVPR). IEEE Computer Society, 2017.

[14] A. Karpathy, G. Toderici, S. Shetty, T. Leung, R. Sukthankar and L. Fei-Fei, "Large-Scale Video Classification with Convolutional Neural Networks," 2014 IEEE Conference on Computer Vision and Pattern Recognition, 2014, pp. 1725–1732, doi: 10.1109/CVPR.2014.223

[15] Bengio Y. Learning Deep Architectures for AI[J]. Foundations & Trends in Machine Learning, 2009, 2(1):1–127.

[16] Russakovsky O, Deng J, Su H, et al. ImageNet Large Scale Visual Recognition Challenge[J]. International Journal of Computer Vision, 2014:1–42.

[17] Du T, Ray J, Shou Z, et al. ConvNet Architecture Search for Spatiotemporal Feature Learning. 2017.

[18] Fernández-Llorca D, Biparva M, Izquierdo-Gonzalo R, et al. Two-Stream Networks for Lane-Change Prediction of Surrounding Vehicles. 2020.

[19] Kumawat S, Verma M, Nakashima Y, et al. Depthwise Spatio-Temporal STFT Convolutional Neural Networks for Human Action Recognition[J]. IEEE Transactions on Pattern Analysis and Machine Intelligence, 2021, PP(99):1–1. 10.1109/TPAMI.2021.3076522 33914681

[20] Sarabu A, Santra A K. Distinct Two-Stream Convolutional Networks for Human Action Recognition in Videos Using Segment-Based Temporal Modeling. 2020.

[21] Hochreiter S, Schmidhuber J. Long Short-Term Memory[J]. Neural Computation, 1997, 9(8):1735–1780. 10.1162/neco.1997.9.8.1735 9377276

[22] Donahue J, Hendricks L A, Guadarrama S, et al. Long-term Recurrent Convolutional Networks for Visual Recognition and Description[J]. Elsevier, 2015. 10.1109/TPAMI.2016.2599174 27608449

[23] Ng Y H, Hausknecht M, Vijayanarasimhan S, et al. Beyond short snippets: Deep networks for video classification[C]// 2015 IEEE Conference on Computer Vision and Pattern Recognition (CVPR). IEEE, 2015.

[24] Sudhakaran S, Lanz O. Learning to Detect Violent Videos using Convolutional Long Short-Term Memory[C]// IEEE. IEEE, 2017:1–6.

[25] Zhenyang Li Kiril, et al. VideoLSTM convolves, attends and flows for action recognition[J]. Computer Vision & Image Understanding Cviu, 2018.

[26] Song S, Lan C, Xing J, et al. An End-to-End Spatio-Temporal Attention Model for Human Action Recognition from Skeleton Data. 2016.

[27] Dai M, Srivastava A. Video-Based Action Recognition Using Dimension Reduction of Deep Covariance Trajectories[C]// 2019 IEEE/CVF Conference on Computer Vision and Pattern Recognition Workshops (CVPRW). IEEE, 2019.

[28] Ahsan U, Sun C, Essa I. DiscrimNet: Semi-Supervised Action Recognition from Videos using Generative Adversarial Networks[J]. 2018.

[29] Carreira J, Zisserman A. Quo Vadis, Action Recognition? A New Model and the Kinetics Dataset[J]. IEEE, 2017.

[30] Laptev I, Marszalek M, Schmid C, et al. Learning Realistic Human Actions from Movies[C]// 2008 IEEE Computer Society Conference on Computer Vision and Pattern Recognition (CVPR 2008), 24–26 June 2008, Anchorage, Alaska, USA. IEEE, 2008.

[31] Yan S, Xiong Y, Lin D. Spatial Temporal Graph Convolutional Networks for Skeleton-Based Action Recognition[J]. 2018.

[32] Al M. LNCS 7065—Sequential Deep Learning for Human Action Recognition.

[33] Le T L, Than C C, Nguyen H Q, et al. Enhancing Multi-stream Graph Convolutional Network for Skeleton-based Human Activity Recognition by Adaptive Model. 2021.

[34] Bobick Aaron F, et al. The Recognition of Human Movement Using Temporal Templates.[J]. IEEE Transactions on Pattern Analysis & Machine Intelligence, 2001.

[35] Liang C, Qi L, He Y, et al. 3D Human Action Recognition Using a Single Depth Feature and Locality-Constrained Affine Subspace Coding[J]. IEEE Transactions on Circuits and Systems for Video Technology, 2017:1–1.

[36] Hu T, Zhu X, Guo W, et al. Human action recognition based on scene semantics[J]. Multimedia Tools and Applications, 2018.

[37] Niebles J C, Chen C W, Li F F. Modeling Temporal Structure of Decomposable Motion Segments for Activity Classification[C]// European Conference on Computer Vision. Springer, Berlin, Heidelberg, 2010.

[38] Du T, Wang H, Torresani L, et al. A Closer Look at Spatiotemporal Convolutions for Action Recognition[J].

[39] Diba A, Fayyaz M, Sharma V, et al. Spatio-Temporal Channel Correlation Networks for Action Classification[J]. Springer, Cham, 2018.

[40] Tran D, Wang H, Torresani L, et al. Video Classification with Channel-Separated Convolutional Networks[J]. 2019.

[41] Feichtenhofer C, Fan H, Malik J, et al. SlowFast Networks for Video Recognition[C]// 2019 IEEE/CVF International Conference on Computer Vision (ICCV). IEEE, 2019.

[42] Feichtenhofer C. X3D: Expanding Architectures for Efficient Video Recognition[J]. IEEE.

[43] Ji J, Krishna R, Li F F, et al. Action Genome: Actions As Compositions of Spatio-Temporal Scene Graphs[J]. IEEE, 2020.

[44] Cao K, Ji J, Cao Z, et al. Few-Shot Video Classification via Temporal Alignment[J]. 2019.

[45] Lotter W, Kreiman G, Cox D. Deep Predictive Coding Networks for Video Prediction and Unsupervised Learning[J]. 2016.

[46] Wang Y, Long M, Wang J, et al. PredRNN: Recurrent neural networks for predictive learning using spatiotemporal LSTMs. 2017. 10.1109/TPAMI.2022.3165153 35380958

[47] Wang Y, Gao Z, Long M, et al. PredRNN++: Towards A Resolution of the Deep-in-Time Dilemma in Spatiotemporal Predictive Learning[J]. 2018.

[48] Zhuang C, She T, Andonian A, et al. Unsupervised Learning from Video with Deep Neural Embeddings[J]. 2019.

[49] Diba A, Fayyaz M, Sharma V, et al. Temporal 3D ConvNets: New Architecture and Transfer Learning for Video Classification[J]. 2017.

[50] Lin J, Gan C, Han S. TSM: Temporal Shift Module for Efficient Video Understanding[J]. 2018. 10.1109/TPAMI.2020.3029799 33035158

[51] Luo C, Yuille A. Grouped Spatial-Temporal Aggregation for Efficient Action Recognition[J]. IEEE, 2019.

[52] Xie S, Girshick R, Dollár P, et al. Aggregated Residual Transformations for Deep Neural Networks[J]. IEEE, 2016.

[53] Zhang X, Zhou X, Lin M, et al. ShuffleNet: An Extremely Efficient Convolutional Neural Network for Mobile Devices[J]. 2017.

